# Dose-Dependent Effects of Cross-Linked Hyaluronic Acid Through Intradermal Injection on Female Facial Photoaging: A Prospective Study

**DOI:** 10.1093/asjof/ojaf046

**Published:** 2025-05-27

**Authors:** Shanqing Wang, Zicong Zhu, Yanting Shih, Yun Zhang, Yong Liao, Xiaoying Chen

## Abstract

**Background:**

Skin photoaging significantly affects skin health and aesthetics, with undesirable appearances negatively impact individual psychological well-being. Intradermal injection of cross-linked hyaluronic acid (HA) has been successfully utilized to improve facial skin photoaging. However, the correlations between the number of treatments and therapeutic efficacy, as well as the dosage and therapeutic outcomes of intradermal injections of cross-linked HA, remain underexplored.

**Objectives:**

The authors observe the effects of intradermal injection of cross-linked HA in different doses and frequencies on improving facial skin photoaging.

**Methods:**

A randomized, split-face design with participant blinding, self-controlled, prospective clinical study was conducted to evaluate the efficacy of different dosages of intradermal injections of cross-linked HA in improving mild-to-moderate female facial photoaging.

**Results:**

Cross-linked HA significantly improved facial photoaging scores, reduced transepidermal water loss, enhanced skin elasticity, and increased patients' satisfaction. The effects were cumulative following sequential treatments, with the high-dosage group demonstrating superior efficacy and longer lasting outcomes compared with the low-dosage group.

**Conclusions:**

High-dosage, multisession intradermal injection protocols of cross-linked HA offer more effective improvements in the manifestations of facial photoaging, with superior efficacy and longer lasting outcomes.

**Level of Evidence: 2 (Therapeutic):**

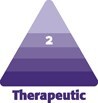

Approximately 80% of extrinsic skin aging is attributed to photoaging, which is primarily caused by prolonged exposure to ultraviolet (UV) radiation, leading to accelerated skin aging. Photoaging results in diminished cellular function and reduced cell counts in the epidermis and dermis, flattening of the basement membrane, and a decline in both the quantity and quality of the extracellular matrix. Consequently, the skin becomes thinner and exhibits wrinkles, laxity, telangiectasia, pigmentation, compromised barrier function, dryness, and, in severe cases, the development of skin tumors.^1-5^ The primary mechanisms of photoaging include oxidative stress-induced collagen degradation, solar elastosis, and chronic inflammatory responses.^6-10^ Skin aging not only affects facial aesthetics but also has negative impacts on the physical and mental well-being of patients, leading to decreased self-confidence, anxiety, and social phobia. Thus, addressing skin aging holds both aesthetic and psychological significance, contributing to improved mental health and overall life satisfaction.

Currently, a wide range of treatments are available for photoaged skin, including medications, chemical peels, lasers, radiofrequency, injections, and surgical interventions.^11^ Hyaluronic acid (HA), an endogenous component of the skin extracellular matrix, possesses exceptional hydration capabilities and plays a critical role in maintaining skin structure and elasticity.^12-17^ In recent years, intradermal injections of HA, particularly those formulated with low degrees of cross-linking for prolonged efficacy, have been widely utilized to improve photoaged skin. Studies have shown that HA with low degrees of cross-linking not only enhances skin appearance through volumization and hydration but also stimulates fibroblasts by increasing local mechanical tension, promoting collagen synthesis, significantly improving skin barrier function and elasticity, and delivering longer time results.^18-22^ This treatment is safe, provides long-lasting effects, requires minimal downtime, and is associated with high patient satisfaction, making it a common approach for facial skin antiaging treatments.

The therapeutic effects of cross-linked HA intradermal injections in relation to treatment frequency and dosage remain inadequately explored. This lack of systematic research results in a limited scientific basis for clinical applications and uncertainty regarding treatment effectiveness. This study aims to investigate the effects of different doses and injection frequencies of cross-linked HA intradermal injections on the improvement of facial photoaging and the duration of its effects.

## METHODS

### Study Design and Ethical Approval

This study was a prospective, split-face, self-controlled clinical trial conducted over 10 months, from January 2024 to October 2024. The study enrolled women aged 26 to 55 years with mild-to-moderate photoaging and equivalent Alexiades photoaging scores on both sides of the face, details of the Alexiades photoaging scores can be referred to in the supplemental material.^23,24^ Each side of the face received injections of cross-linked HA at either a low or a high dose. Sample size estimation using Power Analysis and Sample Size software indicated a requirement of ∼27 half-faces per dose group, corresponding to 27 participants. Considering a 10% dropout rate, 30 participants were planned for recruitment. The study was approved by the Ethics Committee of Ruijin Hospital, Shanghai Jiao Tong University School of Medicine (Ethics Approval No.: 2023-Lunshen-431).

### Subject Selection

Patients were recruited based on the following inclusion and exclusion criteria. Female participants aged 26 to 55 years with mild-to-moderate photoaging according to the Alexiades scoring system (scores of 1-2.5) with equivalent scores on both sides of the face; willing to use effective contraception starting 1 month before and continuing throughout the study; capable of providing written informed consent after detailed explanation; and committed to complying with the study protocol.^23^ Participants with a history of sun exposure within the past 4 weeks; inflammatory or infectious facial skin conditions; severe dermatologic or systemic diseases; use of topical or oral retinoids, steroids, or insulin within the past year; use of corticosteroids, antihistamines, or photosensitizing drugs within the past month; keloid-prone individuals; pregnancy or lactation; history of cosmetic treatments within 6 months; hypersensitivity to lidocaine or HA; unrealistic expectations; or noncompliance with the study protocol.

### Treatment Protocol

Participants received monthly intradermal injections of cross-linked HA with low degrees of cross-linking over 3 consecutive months. One side of the face was randomly assigned to receive a low dose (24 mg), whereas the contralateral side received a high dose (48 mg), maintaining the same dosage in subsequent treatments. Both sides were injected using the same electronic injector and 9-hole needle, with sufficient topical anesthesia applied to minimize the participants’ sensitivity to needle pricks, thereby reducing their ability to perceive differences in injection frequency and density. In this split-face, controlled trial, each participant's left and right facial sides were randomly assigned to receive either the low-dose or high-dose treatment. Randomization was performed using a simple randomization sequence (1:1 ratio) generated in Microsoft Excel. The allocation was concealed in opaque, sealed envelopes until the time of injection. The evaluating physicians remained blinded to the treatment assignments throughout the study. Facial intradermal injections were performed using a digital syringe injector equipped with a disposable 9-hole microneedle (31 G). Each injection delivered a precise dose of 0.0313 mL, with a negative pressure setting of 3 to facilitate optimal dermal diffusion. The injection depth was adjusted according to the treatment area: 0.4 mm for the periorbital region and 0.6 to 0.8 mm for the rest of the face.

Equipment and materials: Digital Syringe Injector (Panace-DS-30, Huons Meditech Co., Ltd, Korea), disposable sterile multineedle injectors (CK-multineedle-9, Contac Korea Corporation, Korea); injectable modified HA gel (Aqua type, Bloomage Biotechnology Corporation Limited); 5% compound lidocaine cream (Beijing Ziguang Pharmaceutical Co., Ltd, China; 10 g, National Drug Standard H20063466); and medical wound dressing (Runzhi Zhenhuo, Bloomage Biotechnology Corporation Limited).

### Evaluation Metrics

#### Alexiades Photoaging Score

After facial cleansing, VISIA imaging (Canfield Scientific) was used to capture standardized frontal and bilateral photographs in a darkroom at baseline, 1 month after each treatment, and 3 and 6 months after the third treatment. Two experienced dermatologists independently assessed the photoaging scores for each side using a blinded evaluation process to ensure impartiality.

#### Transepidermal Water Loss and Skin Elasticity Assessment

Measurements were conducted at baseline, 1 month post each treatment, and at 1, 3, and 6 months after completing the 3 treatment sessions. Transepidermal water loss (TEWL) was evaluated using the Tewameter TM300 (Courage + Khazaka Electronic GmbH, Cologne, Germany), and skin elasticity (*R*^2^ value) was assessed with the Cutometer Dual MPA 580 (Courage + Khazaka Electronic GmbH).

#### FACE-Q Skin Satisfaction Scale

Participants completed FACE-Q questionnaires to evaluate satisfaction with skin condition on both sides of the face at baseline and at 1, 3, and 6 months posttreatment.

### Statistical Analysis

Data were analyzed using IBM SPSS Statistics (IBM Corp., Armonk, NY) and GraphPad Prism (GraphPad Software, San Diego, CA). Nonparametric tests were employed to compare baseline skin metrics between the left and right sides, and to analyze changes in Alexiades scores, skin metrics, and differences between different dosage groups before and after treatments.

## RESULTS

### Baseline Characteristics of Study Subjects

A total of 30 female participants completed all 3 treatment sessions and follow-up assessments. The enrolled participants were aged between 28 and 55 years, and the mean age was 41.47 ± 8.37 years old. Both sides of the face demonstrated equivalent levels of photoaging, with a mean Alexiades photoaging score of 1.78 ± 0.54. At baseline, the TEWL values of the low- and high-dose groups were comparable, at 10.85 ± 0.86 and 10.89 ± 0.83 g/m^2^ h, respectively (*P* = .979). No significant difference was observed in skin elasticity (*R*^2^ values) between the low-dose group (0.4717 ± 0.02) and the high-dose group (0.4725 ± 0.02, *P* > .05). The FACE-Q skin satisfaction score was 39.8 ± 10.36 (Table 1).

**Table 1.** Patients' Characteristics at Baseline

Characteristics	Value
Age, years, (mean ± SD)	41.47 ± 8.37
Female sex, *n* (%)	30 (100)
Alexiades skin aging scale (mean ± SD)	1.78 ± 0.54
FACE-Q score (mean ± SD)	39.8 ± 10.36
TEWL (mean ± SEM)	
Left	10.85 ± 0.86
Right	10.89 ± 0.83
Skin elasticity (*R*^2^) (mean ± SEM)	
Left	0.47 ± 0.02
Right	0.47 ± 0.02

SD, standard deviation; SEM, standard error of the mean; TEWL, transepidermal water loss.

### Comparison of Alexiades Photoaging Scores Before and After Treatment

In the low-dose group, Alexiades photoaging scores significantly decreased 1 month after each treatment session and 3 months after completing the 3 treatment sessions compared with baseline (*P* < .001 for all time points). However, no statistically significant difference was observed at 6 months posttreatment compared with baseline (*P* = .083).

In the high-dose group, the Alexiades photoaging scores significantly decreased at all posttreatment time points compared with baseline (*P* < .001 for all time points).

**Figure 1.**
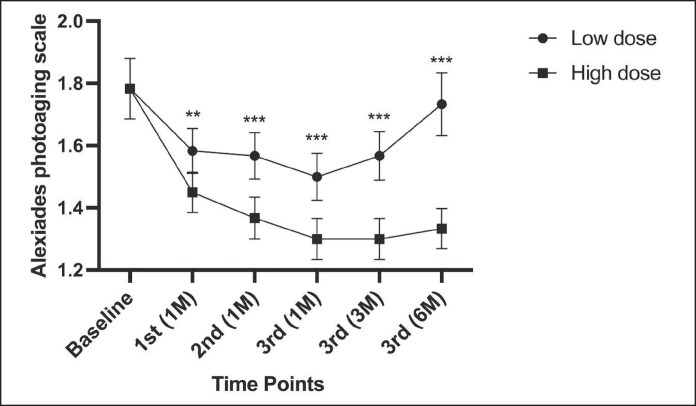
Changes in Alexiades photoaging scores: low-dose group: scores significantly decreased at 1 and 3 months (*P* < .001) but showed no difference from baseline at 6 months (*P* = .083). High-dose group: scores remained significantly lower than baseline at all time points (*P* < .001). Between-group comparison: The high-dose group consistently had lower scores than the low-dose group (*P* ≤ .005). **P < .01; ***P < .001.

**Figure 2.**
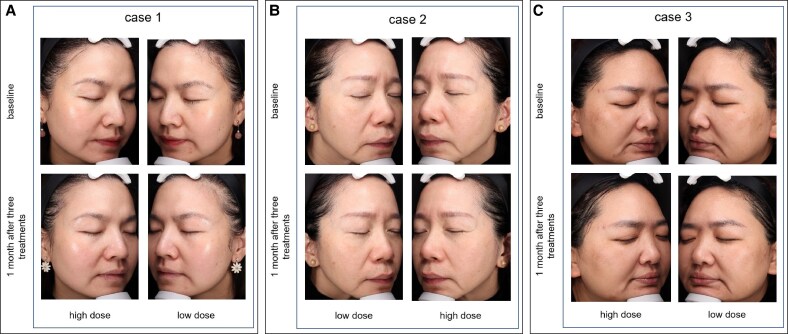
Representative clinical photographs showing skin changes in 3 cases (A) Case 1, female, 43 years old; (B) Case 2, female, 53 years old; (C) Case 3, female, 41 years old at baseline and 1 month after 3 treatments. Photoaging scores improved significantly after 3 treatments, with the high-dose group showing greater improvements compared with the low-dose group.

The high-dose group showed significantly reduced Alexiades photoaging scores compared with the low-dose group at all follow-up time points: 1 month after the first treatment (*P* = .005), 1 month after the second treatment (*P* < .001), 1 month after the third treatment (*P* < .001), and at 3 months (*P* < .001) and 6 months (*P* < .001) posttreatment (Figures 1, 2).

### Comparison of Skin Parameters Before and After Treatment

After each treatment session, TEWL decreased compared with baseline, with progressive reductions observed as the number of treatments increased, maintaining these lower levels until 6 months posttreatment. The high-dose group demonstrated superior improvements compared with the low-dose group.

**Figure 3.**
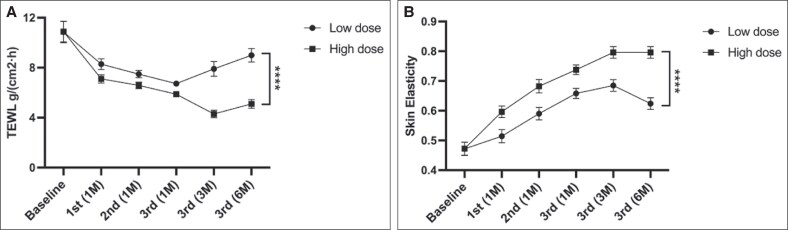
Changes in transepidermal water loss (TEWL) and skin elasticity: (A) TEWL: low-dose group: TEWL significantly decreased at 1 and 3 months posttreatment (*P* < .001) but partially rebounded by 3 months and showed no difference from baseline at 6 months (*P* = .06). High-dose group: TEWL remained significantly lower than baseline at all time points (*P* < .0001). Comparison: TEWL was consistently lower in the high-dose group than in the low-dose group (*P* = .035 to *P* < .0001). (B) Skin elasticity (*R*^2^ value): low-dose group: no significant change after the first treatment (*P* = .4347), but elasticity improved significantly from 1 month postsecond treatment onwards (*P* < .001), with partial regression at 6 months. High-dose group: elasticity improved significantly at all time points (*P* < .0001) and continued to increase at 6 months. Comparison: the high-dose group showed consistently higher elasticity than the low-dose group (*P* = .007 to *P* < .0001).

In the low-dose group, TEWL showed significant reductions at the following time points: 1 month after the first treatment (*P* = .0038), 1 month after the second treatment (*P* < .0001), 1 month after the third treatment (*P* < .0001), and 3 months after completing the 3-treatment regimen (*P* = .0006). TEWL showed a trend toward an increase at 3 months postregimen completion compared with 1 month postregimen completion, although the difference was not statistically significant (*P* = .453). At 6 months posttreatment, TEWL showed no statistically significant difference compared with baseline (*P* = .06). TEWL in the high-dose group was significantly lower than baseline at all follow-ups: 1 month after each treatment session and at 3 and 6 months postregimen completion (all *P* < .0001). Across all posttreatment time points, TEWL values in the high-dose group were significantly lower than those in the low-dose group (*P* = .035, *P* = .027, *P* = .004, *P* < .0001, *P* < .0001; Figure 3).

Skin elasticity increased compared with baseline after treatment, with continued improvements as the number of treatments increased. The high-dose group exhibited greater improvements than the low-dose group.

Following the first treatment, skin elasticity (*R*^2^ value) in the low-dose group showed no significant difference from baseline (*P* = .4347). However, skin elasticity was significantly improved compared with baseline at the following time point: 1 month after the second treatment (*P* = .0003), 1 month after the third treatment (*P* < .0001), and 3 months (*P* < .0001) and 6 months (*P* < .0001) after completing the 3-treatment regimen. By 6 months posttreatment, skin elasticity partially regressed compared with 3 months posttreatment. In the high-dose group, skin elasticity (*R*^2^ value) showed a significant improvement at all posttreatment time points compared with baseline (*P* < .0001 for all time points). Skin elasticity continued to improve until 6 months posttreatment. The *R*^2^ values in the high-dose group were significantly higher than those in the low-dose group at all posttreatment assessments: 1 month after the first treatment: *P* = .007, 1 month after the second treatment: *P* = .004, 1 month after the third treatment: *P* = .001, 3 months postregimen completion: *P* = .0002, 6 months postregimen completion: *P* < .0001 (Figure 3).

### Subjective Satisfaction Scores

Both groups had identical baseline FACE-Q scores (39.80 ± 10.36). At 1 month postthird treatment, the low-dose group (58.67 ± 8.38) scored significantly lower than the high-dose group (65.08 ± 8.87, *P* = .0056). This superiority persisted at 3 months (60.80 ± 8.79 vs 55.43 ± 8.51, *P* = .0194) and 6 months (56.87 ± 8.15 vs 51.70 ± 8.38, *P* = .0186; Table 2).

**Table 2.** Mean FACE-Q Scores (SD)

	Left (low dose)	Right (high dose)	*P*-value
Baseline	39.80 (10.36)	39.80 (10.36)	—
1 month postfinal treatment	58.67 (8.38)	65.08 (8.87)	.0056^a^
3 month postfinal treatment	55.43 (8.51)	60.80 (8.79)	.0194^b^
6 month postfinal treatment	51.70 (8.38)	56.87 (8.15)	.0186^b^

SD, standard deviation.

^a^ *P* < .01

^b^ *P* < .05.

### Adverse Reactions and Safety Evaluation

No treatment-related serious adverse events occurred during the entire trial period. The observed adverse reactions were all transient and reversible. The most common reactions included bruising and erythema at the injection sites, with an incidence of 23.3%. These reactions did not require special treatment and resolved spontaneously within 2 days.

## DISCUSSION

Eighty percent of extrinsic skin aging is attributed to photoaging, which is a process of skin aging induced by UV radiation. This process is accompanied by a reduction in the synthesis of HA in the epidermis and dermis, as well as accelerated degradation, leading to a significant decrease in the total HA content within the skin.^16^ Previous studies have shown that direct injection of low-cross-linked sodium hyaluronate into the dermis can effectively improve skin photoaging.^18-22^ This study further confirms, through a randomized, split-face controlled, prospective clinical trial, that cross-linked HA dermal injection can significantly improve the Alexiades photoaging score, reduce TEWL, and increase skin elasticity. Furthermore, the therapeutic effect accumulates with the number of treatments. After 3 cumulative treatments, the effect can last for up to 6 months, which aligns with the degradation time of this kind of cross-linked HA (3-6 months).^25,26^

This study found that the high-dose group exhibited significantly better results at all time points compared with the low-dose group, with a longer duration of effect, indicating a potential positive correlation between dosage and clinical efficacy. High-dose HA injections not only significantly improve the appearance of photoaged skin but also continuously enhance skin function. Previous literature reports that cross-linked HA dermal injections can stimulate fibroblast proliferation and promote collagen synthesis.^19,20^ This finding is consistent with the increase in skin elasticity observed in our study after treatment. Additionally, our study further confirms the positive correlation between treatment frequency, dosage, and efficacy, highlighting the crucial role of sustained treatment and an appropriate dosage in improving photoaged skin.

The physicochemical properties of cross-linked HA products are closely related to their HA concentration and degree of cross-linking.^27-30^ Higher concentrations and cross-linking degrees result in greater cohesiveness and elasticity, whereas lower concentrations and cross-linking degrees yield the opposite. Low-concentration, low-cross-linked HA has lower cohesiveness and elasticity, making it particularly suitable for facial skin because of its softness and ability to withstand higher shear forces.^31^ The low-cross-linked HA used in this study (0.6% cross-linking, 12 mg/mL HA content) demonstrated good safety in dermal injection treatments, with no significant adverse reactions observed. These results suggest that this low-cross-linked HA is safe for use in skin antiaging treatments, particularly for skin photoaging. Moreover, the standardized full-face injection of 72 mg of cross-linked HA (6 mL at 12 mg/mL) demonstrated a favorable safety profile, with no significant adverse events reported in any of the 30 patients. Additionally, it exhibited clinically meaningful efficacy, as evidenced by significant improvements in TEWL, skin elasticity, and patient satisfaction scores in this study.

Based on the findings of this study, it is recommended to use multiple high-dose cross-linked HA injection treatments for skin photoaging to achieve superior and lasting clinical outcomes.

In this study, a relatively traditional injection interval of once per month was adopted. Given the prolonged degradation time of cross-linked HA, there remains room for optimization in determining the ideal injection interval. Additionally, the impact of single-injection dose selection and injection depth on the improvement of photoaging requires further investigation. The study participants included individuals with mild-to-moderate photoaging. For those with severe photoaging (eg, deep wrinkles and significant skin laxity), a total dose of 72 mg may be insufficient to achieve visible morphological improvement. Therefore, exploring higher doses or combination treatment strategies, such as incorporating energy-based devices, may be necessary. Although the 72 mg total dose demonstrated both safety and efficacy, a dose–response curve correlating the injected amount with clinical improvement has not yet been established. For instance, higher doses (eg, 90-120 mg) may further enhance dermal structural support, thereby improving skin elasticity. However, their safety—particularly the risk of localized protrusions—requires careful evaluation. Future studies could employ gradient dose groups or adaptive trial designs to determine the optimal dosage range.

Although participants were blinded to dose allocation, the unilateral injection volume difference (2 vs 4 mL) could potentially affect blinding maintenance through differences in posttreatment facial fullness or injection frequency and density. However, postinjection adverse reaction questionnaires indicated that no participants reported perceptible asymmetry, and no significant differences in facial erythema were observed, suggesting the feasibility and effectiveness of the blinding approach in this study. Besides, the sample size in this study was relatively small (only 30 participants with photoaged skin). Therefore, further studies with larger sample sizes and multicenter trials are needed to validate the efficacy and safety of this treatment regimen. Additionally, long-term follow-up studies are necessary to evaluate the long-term effects and potential safety concerns of low-concentration HA with low degrees of cross-linking dermal injections.

## CONCLUSIONS

Low-concentration HA with low degrees of cross-linking significantly improves the Alexiades photoaging score, enhances skin TEWL, skin elasticity, and patient satisfaction. A high-dose, multiple-injection regimen yields better results with a longer duration of clinical effect, and participants report higher satisfaction.

## Supplementary Material

ojaf046_Supplementary_Data
