# Supplementary material for: Dose-Dependent Effects of Cross-Linked Hyaluronic Acid Through Intradermal Injection on Female Facial Photoaging: A Prospective Study
Source: Aesthet Surg J Open Forum. 2025 May 27;7:ojaf046. doi: 10.1093/asjof/ojaf046 (PMC12212054; doi:10.1093/asjof/ojaf046)
Supplement: ojaf046_Supplementary_Data [file ojaf046_supplementary_data.docx]

**Supplementary Table**: Alexiades photoaging scores

|  |  | Categories of Skin Aging and Photodamage | | | | | | |
| --- | --- | --- | --- | --- | --- | --- | --- | --- |
| Grading Scale | Descriptive Parameter | Rhytides | Laxity | Elastosis | Dyschromia | Erythema-Telangiectasia(E-T) | Keratoses | Texture |
| 0 | None | None | None | None | None | None | None | None |
| 1 | mild | Wrinkles in motion, few, superficial | Localized to nasolabial(nl) folds | Early, minimal yellow hue | Few(1-3) discrete small(<5mm) lentigines | Pink E or few T, localized to single site | Few | Subtle irregularity |
| 1.5 | mild | Wrinkles in motion, multiple, superficial | Localized, nl and early melolabial1, (ml)folds | Yellow hue or early, localized periorbital(po) elastotic beads(eb) | Several(3-6) discrete small lentigines | Pink E or several T localized 2 sites | Several | Mild irregularity in few areas |
| 2 | moderate | Wrinkles at rest, few, localized, superficial | Localized nl/ml folds, early jowels, early submental/sub-mandibular(sm) | Yellow hue, localized po eb | Multiple(7-10), small lentigines | Red E or multiple T loalized to 2 sites | Multiple, small | Rough in few, localized sites |
| 2.5 | moderate | Wrinkles at rest, multiple, localized, superficial | Localized, prominet nl/ml folds, jowels and sm | Yellow hue, po and malar eb | Multiple, small and few large lentigines | Red E or multiple T, localized to 3 sites | Multiple, large | Rough in several, localized areas |
| 3 | advanced | Wrinkles at rest, multiple, forehead, periorbital and perioral sites, superficial | Prominent nl/ml folds, jowels and sm, early nieck strands | Yellow hue, eb involving po, malar and other sites | Many(10-20) small and large lentigines | Violaceous E or many T, multiple sites | Many | Rough in multiples, localized sites |
| 3.5 | advanced | Winkles at rest, multiples, generalized, superficial; few, deep | Deep nl/ml folds, prominent jowels and sm, prominet neck strands | Deep yellow hue, extensive eb with little uninvolves skin | Numerous(>20) or multiple large with little uninvolves skin | Violaceous E, numerous T little uninvolved skin | Little uninvolved skin | Mostly rough little uninvilved skin |
| 4 | severe | Wrinkles throughout, numerous, extensively distributed, deep | Marked nl/ml folds, jowels and sm, neck redundancy and strands | Deep yellow hue, eb throughout, comedones | Numerous, extensive, no uninvolved skin | Deep, violaceous E, numerous thoughout | No uninvolved skin | Rough throughout |
